# Assessment of Compliance with Animal Welfare Requirements Across Poultry Species and Production Categories

**DOI:** 10.3390/ani16050834

**Published:** 2026-03-07

**Authors:** Eva Justova, Vladimir Vecerek, Zbynek Semerad, Marijana Vucinic, Eva Voslarova

**Affiliations:** 1 Department of Animal Protection and Welfare and Veterinary Public Health, Faculty of Veterinary Hygiene and Ecology, University of Veterinary Sciences Brno, 61242 Brno, Czech Republic; justovae@vfu.cz (E.J.); vecerekv@vfu.cz (V.V.); 2 Central Veterinary Administration of the State Veterinary Administration, 12000 Prague, Czech Republic; z.semerad@svscr.cz; 3 Department of Animal Hygiene, Faculty of Veterinary Medicine, University of Belgrade, 11000 Belgrade, Serbia; vucinicm@vet.bg.ac.rs

**Keywords:** animal welfare, domestic chicken, turkey, duck, goose

## Abstract

Animal welfare is a key component of sustainable poultry production and is routinely monitored through official veterinary inspections. The aim of this study was to determine the level of welfare compliance among different poultry species and production categories, to compare compliance levels across these groups, and to assess long-term trends using official inspection data. The study was based on the results of supervisory inspections conducted by veterinary inspectors in poultry farms in the Czech Republic between 2016 and 2024. Welfare compliance was evaluated in laying hens, broiler chickens, turkeys, ducks, and geese using a standardized system of welfare assessment checkpoints applied during official controls. Inspections were classified as compliant or non-compliant based on the presence or absence of deficiencies, and overall compliance levels were calculated as the proportion of animals kept in farms with compliant inspections. Across the entire study period, the proportion of poultry kept in farms with compliant inspections ranged from 82.8% to 98.4%, with the highest compliance level observed in turkeys, followed by ducks and broiler chickens, while the lowest compliance level was recorded in geese. Differences among poultry species and categories were statistically significant (*p* < 0.05). Comparison of two time periods (2016–2018 and 2022–2024) revealed significant improvements in compliance for broiler chickens, ducks, and geese, whereas significant declines were observed for laying hens and turkeys (*p* < 0.001). These results demonstrate clear differences in welfare compliance among poultry species and categories and indicate that compliance trends over time are not uniform across the poultry sector. Official veterinary inspection data provide a valuable tool for large-scale assessment of welfare compliance and for identifying poultry sectors that may benefit from targeted welfare improvement measures.

## Simple Summary

Animal welfare is an important part of poultry farming and is closely linked to animal health, food quality, and public expectations. Poultry farms are regularly inspected by veterinary authorities to ensure that animals are kept under acceptable conditions. In this study, we analyzed the results of these official inspections to evaluate how well different types of poultry farms complied with animal welfare requirements in the Czech Republic over a nine-year period. We focused on five types of poultry: laying hens, broiler chickens, turkeys, ducks, and geese. The aim was to compare the proportion of birds kept in farms that met welfare requirements and to examine how this changed over time. The results showed that most poultry were kept in farms that complied with welfare requirements, but clear differences existed between species. Turkeys and ducks had the highest levels of compliance, while geese had the lowest. Over time, compliance improved in broiler chickens, ducks, and geese, but declined in laying hens and turkeys. These findings show that compliance with welfare requirements does not develop in the same way across all poultry sectors. The study demonstrates that official inspection data can help identify poultry sectors that may need greater attention to further improve animal welfare, benefiting animals, farmers, and society.

## 1. Introduction

Animal welfare is a fundamental component of sustainable poultry production and an important aspect of animal health, food safety, and societal acceptance of livestock farming [1,2]. In poultry production systems, animal welfare is influenced by a wide range of factors, including housing conditions, management practices, environmental parameters, and stockperson competence (e.g., [3,4,5,6,7]). Ensuring appropriate welfare conditions is therefore a key objective of both poultry producers and regulatory authorities [8].

In many countries, including those of the European Union, minimum standards for the protection of poultry are defined by legislation and enforced through official veterinary controls. These inspections are designed to verify compliance with established welfare requirements and to identify deficiencies that may compromise animal well-being [9,10,11,12]. Data generated through official inspection systems represent a valuable source of information for evaluating compliance with animal welfare legislation at the population level, as they are collected using standardized procedures and cover a broad range of production systems and species [13,14].

Poultry production encompasses a variety of species and production categories, such as laying hens, broiler chickens, turkeys, ducks, and geese, which differ markedly in their biology, housing systems, and management practices [15,16,17]. These differences may result in variability in welfare outcomes and in the ability of farms to comply with welfare requirements [18,19]. Comparative assessments of compliance levels among poultry species and categories are therefore essential for identifying sectors with higher or lower adherence to welfare standards and for targeting improvement measures effectively.

In addition to cross-sectional comparisons, the evaluation of compliance trends over time is important for assessing the impact of regulatory measures, technological developments, and changes in production practices. Long-term analyses based on official inspection data can provide insight into whether compliance with welfare requirements is improving, remaining stable, or deteriorating in specific poultry sectors. Such information is particularly relevant for competent authorities and policymakers responsible for animal welfare oversight.

The Czech Republic has a well-established system of official veterinary supervision of animal welfare, carried out by the State Veterinary Administration through regular inspections of poultry farms. However, comprehensive analyses comparing compliance with welfare requirements among different poultry species and production categories and evaluating temporal trends based on inspection outcomes remain limited [8,20].

Therefore, the aim of this study was to determine the overall level of welfare compliance among different poultry species and production categories in the Czech Republic based on official veterinary inspection data, to compare compliance levels across these groups, and to assess long-term trends in compliance over the period from 2016 to 2024.

## 2. Materials and Methods

The study is based on the results of official supervisory inspections conducted by veterinary inspectors in poultry farms between 2016 and 2024. In the Czech Republic, compliance with animal welfare legislation is supervised by the State Veterinary Administration, which performs official controls through regional veterinary authorities. The inspection protocols remained consistent throughout the study period (2016–2024). A major revision of the protocols and the inspection data recording system was implemented prior to 2016, ensuring standardized procedures for the analyzed period. No significant legislative changes affecting poultry production occurred during 2016–2024; the next notable change, the planned ban on enriched cage systems for laying hens, is scheduled for 2027.

Welfare compliance was evaluated in the following species and production categories: laying hens, broiler chickens, turkeys, ducks, and geese. It should be noted that official veterinary inspections primarily evaluate compliance with resource- and management-based animal welfare requirements, rather than direct animal-based measures. Therefore, the reported compliance levels reflect adherence to statutory welfare standards, rather than a direct assessment of animal well-being.

During the period 2016–2024, approximately 112 laying hen, 326 broiler chicken, 63 turkey, 143 duck, and 29 goose farmers were included in the dataset (large producers may operate multiple farms). Inspections were routine, carried out according to the annual activity plan of veterinary farm controls, which incorporates risk evaluation. Complaint-driven inspections in poultry farms do not occur, as farms are not publicly accessible due to strict biosecurity measures. The same farm could be inspected in multiple years, as inspections are repeated periodically.

Inspections were carried out in accordance with the system of welfare assessment checkpoints used by official veterinary authorities during animal farm inspections. The assessment included, in particular:

- Buildings and housing: facilities, interior housing equipment, animal areas, corridors, floors, walls and partitions, disinfection, disinsection, deratization, manure removal, type of housing, group or individual housing, freedom of movement, feeding and watering.
- Zoo hygiene conditions: temperature, humidity, lighting, ventilation, noise, ammonia concentration, and carbon dioxide concentration.
- Outdoor areas: availability and condition of runs.
- Feed and feeding systems; water and watering systems.
- Animal identification and record keeping.
- Animal handling and care: handling procedures, animal management, interventions performed on animals, animal body condition, health status, compliance with minimum welfare standards, personnel competence, record keeping, and other relevant aspects.

For each inspection, farms were categorized as either: compliant—no deficiencies detected in any inspection checkpoint; or non-compliant—at least one deficiency recorded at any checkpoint.

For each species and production category, the number of animals present on inspected farms and the number of animals on farms classified as compliant were determined. The overall welfare compliance level was calculated as the proportion of animals on farms with compliant inspections relative to the total number of inspected animals (relative frequency). This indicator was used as a measure of compliance with animal welfare requirements for each poultry species and category. An animal-based approach was chosen to reflect the actual number of birds affected by compliance or non-compliance, rather than treating each farm equally regardless of flock size. Using animal numbers provides a more policy-relevant measure of welfare outcomes at the population level.

A comparative assessment among species and categories was conducted by statistically comparing the relative frequencies of animals on compliant farms. The aim was to determine which poultry species and categories showed higher or lower levels of compliance with animal welfare legislation.

Temporal trends in compliance were determined by statistically comparing the relative frequencies of animals in compliant farms between two periods: 2016–2018 (Period I) and 2022–2024 (Period II). This comparison allowed evaluation of whether compliance levels for each species and category increased or decreased over time. For the purpose of this analysis, only Period I (2016–2018) and Period II (2022–2024) were included. The intermediate years (2019–2021) were excluded to ensure that the compared periods did not overlap, allowing clear detection of changes over time and avoiding partial attribution of gradual changes to both periods, which could obscure trends.

Data analysis was conducted in Unistat v. 6.5 (Unistat Ltd., London, UK). Differences in relative frequencies were evaluated using the chi-square test, with statistical significance defined as *p* < 0.05.

## 3. Results

**Figure 1:**
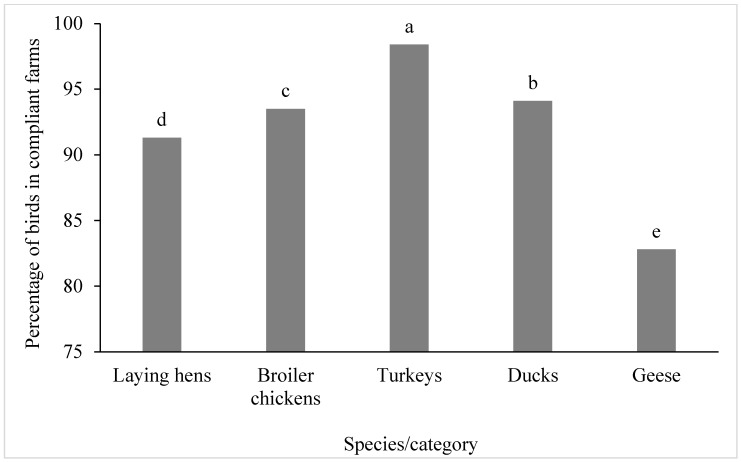
Comparison of relative numbers of poultry in farms with compliant inspections from 2016 to 2024 among poultry species and categories. ^a–e^ Percentages in columns with different letters differ significantly at *p* < 0.05.

The numbers of poultry in the categories laying hens, broiler chickens, turkeys, ducks, and geese in inspected farms during the monitored period are presented in Table 1. The numbers of poultry in farms classified as compliant are shown in Table 2. A comparison of the relative proportions of poultry kept on farms with compliant inspections among individual species and categories over the entire study period from 2016 to 2024 is presented in Figure 1. The results indicate that the proportion of poultry kept on farms with compliant inspections ranged from 82.8% to 98.4% across species and categories. The highest compliance level was observed in turkeys (98.4%), followed by ducks (94.1%), broiler chickens (93.5%), laying hens (91.3%), while the lowest compliance level was recorded in geese (82.8%). Differences among poultry species and production categories were statistically significant (*p* < 0.05).

**Figure 2:**
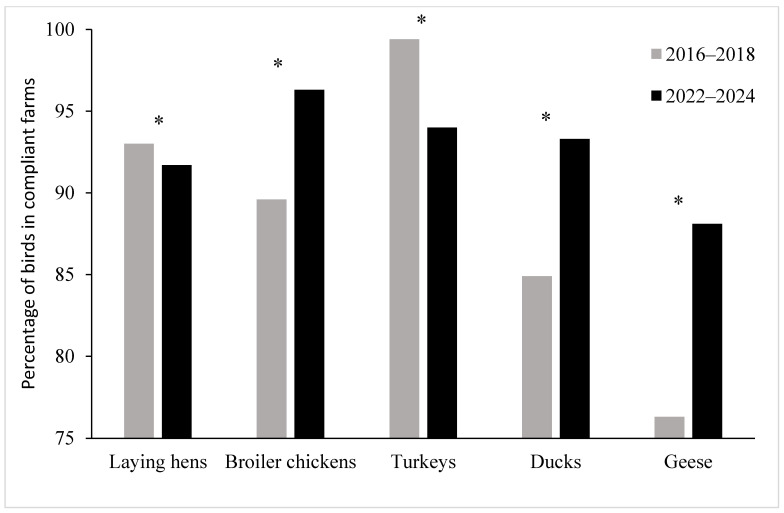
Comparison of the numbers of poultry in farms with compliant inspections between the first period (2016–2018) and the last period (2022–2024). * Percentages differ significantly between the two periods at *p* < 0.001.

A comparison of the relative proportions of poultry kept on farms with compliant inspections for individual species and categories between Period I (2016–2018) and Period II (2022–2024) is shown in Figure 2. The results demonstrate a statistically significant decrease in the proportion of poultry kept on compliant farms for laying hens (*p* < 0.001) and turkeys (*p* < 0.001), while a statistically significant increase was observed for broiler chickens (*p* < 0.001), ducks (*p* < 0.001), and geese (*p* < 0.001) between the two periods.

## 4. Discussion

The present study provides a comprehensive comparison of welfare compliance among different poultry species and production categories based on the outcomes of official veterinary inspections conducted over a nine-year period. The results demonstrate that, overall, a high proportion of poultry were kept on farms compliant with established animal welfare requirements; however, statistically significant differences were observed among species and categories, as well as notable temporal trends. It should be noted that animal welfare legislation generally defines minimum acceptable standards rather than optimal welfare conditions. Consequently, compliance with legal requirements does not necessarily imply a high level of animal welfare, but rather indicates that welfare conditions meet the minimum threshold considered acceptable by current regulations [8].

Across the entire study period, the highest compliance level was observed in turkeys, followed by ducks and broiler chickens, whereas laying hens showed lower compliance and geese exhibited the lowest compliance level. These inter-species differences likely reflect variations in housing systems, management practices, and the degree of standardization and regulatory oversight across poultry sectors. In addition, variation in farm size, sector organization, and the intensity or focus of inspections may also contribute to differences in compliance rates among species and production categories. Turkey and broiler production are typically characterized by intensive systems with clearly defined welfare requirements and standardized management procedures, which may facilitate compliance during official inspections. Production systems for meat birds (chickens, turkeys, and ducks) are generally similar in terms of housing design and management, although species-specific differences exist related to growth rate and production cycle length [18]. Comparable large-scale inspection-based studies are scarce; however, Mullan et al. [20], who evaluated on-farm welfare outcome measures in the United Kingdom, reported that a greater proportion of broiler farms consistently ranked in the best welfare quartile than in the worst quartile, supporting the relatively favorable compliance outcomes observed in broiler production.

In contrast, goose production is typically less intensive, more heterogeneous, and often involves outdoor access, which may increase the likelihood of non-compliance with specific welfare checkpoints. Goose production systems include extensive, intensive, littered-floor, and alternative systems such as free-range and organic production, and the production system represents a key non-genetic factor influencing welfare and behavioral traits in poultry [21]. Geese reared outdoors generally do not require complex or costly housing infrastructure [22], which may lead some producers to place less emphasis on housing-related welfare measures. In the Czech Republic, domestic geese are kept exclusively for meat production, as force-feeding for foie gras production is prohibited by law.

The relatively lower welfare compliance observed in laying hens compared with broiler chickens may be linked to the structural and management complexity of layer housing systems, particularly alternative systems incorporating perches, nests, and litter areas. Although these systems are designed to enhance behavioral expression, they also pose challenges in maintaining uniform environmental conditions, hygiene, and flock management, which are commonly assessed during welfare inspections. A comprehensive review by Schwean-Lardner and Herwig [23] reported that free-run housing systems allow laying hens to express a wider range of natural behaviors and may contribute to improved bone strength; however, these systems are also associated with increased risks of severe feather pecking, cannibalism, smothering, and higher mortality. Laying hens in non-cage systems have been shown to exhibit higher incidences of bone fractures, footpad dermatitis, and bumblefoot, as well as poorer air quality compared with hens housed in furnished cages. Even under well-managed conditions with regular manure removal, ammonia concentrations in free-run systems were reported to be more than twice those observed in cage systems [24]. Furthermore, Vecerkova et al. [19] documented substantially higher numbers of patho-anatomical findings in laying hens at postmortem inspection compared with broiler chickens and turkeys. Similarly, Vecerek et al. [25] reported significantly poorer health status in laying hens, particularly with respect to liver lesions and limb-related injuries, compared with broiler chickens and turkeys.

Analysis of temporal trends revealed divergent developments among poultry species and categories. A statistically significant increase in compliance was observed in broiler chickens, ducks, and geese between the periods 2016–2018 and 2022–2024. These positive trends may reflect increased awareness of animal welfare requirements, improvements in housing technology, or more effective implementation of corrective measures following inspections. In addition, gradual adaptation of producers to evolving welfare legislation and inspection criteria may have contributed to higher compliance rates over time.

Conversely, a statistically significant decrease in compliance was detected in laying hens and turkeys. In laying hens, this decrease may be associated with ongoing structural changes in the sector, including transitions between housing systems and increased production pressures, which can temporarily affect welfare compliance. In the Czech Republic, the majority of laying hens were housed in cage systems during the monitored period, with conventional cages replaced by furnished cages prior to their ban in 2012. However, a gradual transition toward alternative housing systems has been underway, driven both by the planned national ban on cage systems for laying hens from 2027, approved by the Czech Parliament in 2020 [26], and by increasing pressure from commercial retailers. Although conventional cage systems are widely recognized as compromising animal welfare, the optimal direction for alternative housing systems remains under debate [23], as each system presents distinct advantages and limitations [27]. While free-range systems may entail certain welfare risks, Hampel et al. [26] suggest that large-scale adoption of such systems could stimulate the development of new breeding goals aimed at simultaneously improving welfare and production efficiency.

In turkeys, despite their overall high compliance level, the observed decrease suggests that maintaining consistently high compliance remains challenging and may require targeted attention to specific welfare indicators assessed during inspections. Lisiowska et al. [10], who evaluated the effectiveness of veterinary inspection measures in Poland, concluded that undesirable welfare outcomes may be reduced by intensifying control measures applied by veterinary authorities. In this context, continuous and harmonized training of inspectors appears to be essential for improving the effectiveness and consistency of welfare supervision.

It should be emphasized that the assessment in this study was based on the outcomes of official veterinary controls, which classify inspections dichotomously as compliant or non-compliant. While this approach provides robust and standardized data suitable for large-scale comparisons, it does not capture the severity or frequency of individual welfare deficiencies. Nevertheless, the use of animal numbers rather than farm counts enhances the relevance of the findings by reflecting the actual proportion of animals kept under compliant or non-compliant conditions. Therefore, the results should be interpreted as indicators of regulatory compliance rather than comprehensive measures of overall animal welfare quality.

During the study period (2016–2024), inspection protocols and data recording procedures remained formally consistent, and no major legislative amendments affecting poultry production were implemented. This institutional stability supports the comparability of compliance outcomes over time. However, even in the absence of formal regulatory changes, recorded compliance trends may still be influenced by factors such as variation in inspection frequency, shifts in inspection focus toward particular welfare indicators, differences in enforcement stringency, or ongoing training and harmonization of inspectors. Such procedural and organizational factors could affect the likelihood of detecting and recording non-compliance independently of actual changes in farm-level management. These considerations should therefore be taken into account when interpreting longitudinal developments in compliance rates.

## 5. Conclusions

The results highlight clear differences in animal welfare compliance among poultry species and production categories and demonstrate that compliance trends over time are not uniform across the poultry sector. These findings underline the importance of species- and category-specific approaches to welfare monitoring and targeted improvement measures. Continued systematic evaluation of official inspection data represents a valuable tool for identifying areas requiring increased attention and for supporting evidence-based strategies aimed at strengthening compliance with statutory animal welfare requirements in poultry production systems.

## Figures and Tables

**Table 1:** Numbers of poultry in inspected farms.

Species/Category	Period I	Period II	Total ^1^
	2016 to 2018	2022 to 2024	2016 to 2024
Laying hens	7,074,448	9,053,646	25,572,713
Broiler chickens	10,457,836	11,146,264	32,075,476
Turkeys	62,856	22,574	167,560
Ducks	44,088	200,899	358,802
Geese	2450	10,195	15,353

^1^ Total numbers include all years from 2016 to 2024, including the intermediate years 2019–2021.

**Table 2:** Numbers of poultry in farms with compliant inspections.

Species/Category	Period I	Period II	Total ^1^
	2016 to 2018	2022 to 2024	2016 to 2024
Laying hens	6,579,724	8,305,945	23,348,329
Broiler chickens	9,373,568	10,732,147	29,986,256
Turkeys	62,508	21,225	164,877
Ducks	37,432	187,488	337,628
Geese	1870	8986	12,708

^1^ Total numbers include all years from 2016 to 2024, including the intermediate years 2019–2021.

## Data Availability

The original contributions presented in this study are included in the article. Further inquiries can be directed to the corresponding author.
